# The role of religious diversity in social progress

**DOI:** 10.1177/14687968221085615

**Published:** 2022-08

**Authors:** Grace Davie

**Affiliations:** Emeritus, 3286 University of Exeter, UK

**Keywords:** Religion, religious diversity, social progress, International Panel on Social Progress, multiculturalism, secularism, COVID-19

## Abstract

This article brings together the notions of religious diversity and social progress and argues, against the sceptics, that the former can – and indeed must – contribute positively to the latter. To do this, it builds on to a major initiative in which the author had co-responsibility for the material on religion. This was the International Panel on Social Progress (IPSP) which assessed state-of-the-art knowledge that bears on social progress across a wide range of economic, political and cultural questions. The work of the IPSP as a whole is briefly outlined; the article then looks at the chapter on religion within this, foregrounding the material on religious diversity. This material is placed in a wider discussion of multiculturalism and secularism, in which links are made with the work of Tariq Modood and the Bristol Centre for the Study of Ethnicity and Citizenship. A short postscript introduces a more topical issue. It considers the role of religious communities (more especially religious minorities) as societies confront the ravages of COVID-19.

## Introduction

The following article brings together the notions of religious diversity and social progress and argues, against the sceptics, that the former can – and indeed must – contribute positively to the latter. To do this, it builds on to a major initiative in which I had co-responsibility for the material on religion. This was the International Panel on Social Progress (IPSP) which came into existence to assess the state-of-the-art knowledge that bears on social progress across a wide range of economic, political and cultural questions.^1^ The overall goal was to provide the target audience (individuals, movements, organizations, politicians, decision-makers and practitioners) with the best expertise that social science can offer in this field. A three-volume report was published by CUP in 2018 entitled *Rethinking Society for the 21st Century* (International Panel on Social Progress, 2018).^2^

Volume 3 contains a chapter of some 30,000 words dedicated to the place of religion in this enterprise. The present article draws on this chapter in two ways: first on the conceptualization of the key terms (religion on the one hand and social progress on the other); and second on the data and arguments that relate both to religion per se and to religious diversity. With regard to the latter, the article looks first at the existence of religious diversity across the globe and then at its management. Both elements are then considered in light of wider debates in the field, in a section which foregrounds Tariq Modood’s discussion of both multiculturalism and secularism and the continuing work of the Bristol Centre for the Study of Ethnicity and Citizenship, the focus of this special issue.

A short postscript introduces a more topical issue. It considers the role of religious communities (more especially religious minorities) as societies confront the ravages of a global pandemic: COVID-19. The material is necessarily preliminary but demonstrates the potential of faith communities, including minorities, to contribute positively to an unprecedented situation, a challenge that will merit close and continued attention for the foreseeable future.

### The International Panel on Social Progress

A full discussion of the International Panel on Social Progress lies beyond this article. Some details are however necessary, notably the skillsets of the team assembled to address the place of religion within this undertaking. This was built to ensure – as far as possible – expertise in all global regions, in the major world faiths, and in the different branches of social science. Above all, it was necessary to find scholars who had hands-on experience in researching religion and religious issues in the field who would be able to locate and interpret the most useful sources of data.^3^

Their first and most demanding challenge became clear very quickly and is as central to this article as it was to the IPSP chapter: that is the realization that significant sections of the social-scientific community – including a number of colleagues in the IPSP – were and remain hesitant about the relationship between religion and social progress as we were learning to understand this (see below). This hesitancy took two forms: either religion was irrelevant (i.e. no longer of significance), or it was negatively perceived – in other words, inimical to social progress. The fact that religion was (or more accurately was deemed to be) ‘back’ was therefore a problem. Put differently, if it is clear that there has been a marked shift in attitudes amongst those who work in the field of religion regarding the place of religion in the modern world, this is much less evident in the mainstreams of the social sciences, where what might be termed Enlightenment paradigms – the assumption that modernization necessarily implies secularization – still persist. It followed that a chapter on religion in a report concerned with the social progress was seen almost as an oxymoron. Correcting this misapprehension, therefore, becomes central to our task.

A final – and related – point concludes this section: that is to clarify what is meant first by religion and then by social progress. With regard to the former, escaping the limitations of a purely Western (primarily European) perspective was the first step. Religion is more – much more – than the broad range of institutions and beliefs traditionally recognized by social science. It is rather a lived, situated and constantly changing reality, that has as much to do with navigating everyday life as it does with the supernatural. It follows that secularity (meaning here the absence of religion) should be considered an equally fluid entity, whose distinction from religion will vary from place to place or institution to institution – a division decided more by the context in question that by a pre-determined essence.

Understanding social progress – clearly a crucial issue for the IPSP as a whole – required a similar shift in perspective: the assumption that progress is somehow built into history must be set on one side. The consortium deployed instead the notion of a compass, seeing this as a helpful metaphor in the sense that it sets the line of travel, but recognizes that the map in question is complex and the destination elusive. What is considered progress in one situation may be differently assessed in another. The argument is set out in detail in Chapter 2 of the final report and merits very careful attention (Richardson and Schokkaert et al., 2018). What then is the relevance of religion – and in particular religious diversity – in this narrative?

### The chapter on religion

The chapter – entitled ‘Religions and Social Progress: Critical Assessments and Creative Partnerships’ – starts from the fact that more than eight-in-ten of the world’s population affirms some kind of religious identification, a proportion that is growing rather than declining, pointing the reader to the statistics compiled by the Pew Forum and freely available on their website.^4^ This work is continually updated, noting both stabilities and fluctuations, and offering explanations for both. The literature is carefully sifted. Differential birthrates are particularly striking (Pew Research Centre, 2017) to the advantage of religious groups (notably the Muslim population) rather than the unaffiliated. In global terms, the low point of secularity is found in the 1970s. It follows that paying close attention to religion is, and will remain, essential in global debates including those that relate to social progress.

Keeping this in mind, the chapter team looked systematically at the significance of belief and practice in everyday lives and local contexts. To get a handle on a mass of data, the impact of religion and its relevance to social progress was analysed across a carefully selected range of fields. These included family, gender and sexuality; diversity and democracy; conflict and peace-making; everyday wellbeing; and ecological change. What emerged was an ascending scale, working from the micro to the macro, looking across a variety of economic, political and social institutions, and wherever possible deploying a comparative perspective.

It is important to keep in mind the multiple variables that make up religion. Researchers and policy makers who are interested in social progress should pay careful attention to the following: the power of religious ideas to motivate; the effectiveness of religious practices in shaping ways of life; the potential of religious communities in mobilizing and extending the reach of social change; and the role of religious leaders and symbols in legitimating calls to action. Taken together these attributes add up to a powerful and mobilizing force that can be put to either good or ill. Both are possible, for which reason broad generalizations are rarely helpful; the assessment of particular religions in specific contexts becomes the best way forward.

One example will suffice: to ask whether religion – or certain forms of religion – cause conflict or violence is not the most helpful approach. Much more constructive are enquiries that look systematically at the circumstances in which a violent outcome is likely – a quintessentially social-scientific task demanding the careful scrutiny of multiple factors both singly and in clusters. Contestation over physical spaces turns out to be a likely flashpoint, as does an excess of regulation which leads all too often to negative consequences for religious minorities. Even more important is the evidence that weak or failed states (and the fragile economies associated with them) encourage – by default – violent and authoritarian attempts to restore order, irrespective of any religious factors that may, or may not, be present.

Chapter teams were invited to make concrete recommendations for policy. In our case, these emerged in the form of five interconnected themes that cross cut the data presented. The first underlined the persistence of religion in the twenty-first century; religion is neither vanishing, nor resurgent. The second emphasized the importance of context in discerning outcomes; one size does not fit all. The third stressed the need for cultural competence (i.e. both knowledge and sensitivity) relative to religion in a multiplicity of fora, a statement with huge implications for training. The fourth recognized the significance of religion in initiating change; it is a powerful motivator. The fifth underlined the benefits of carefully chosen partnerships, more often than not between religious and secular agencies. Taken together there is a continuing need for assessment and – where necessary – constructive criticism of both religious ideas and religious actors, bearing in mind that social progress not only evolves but looks different in different places. Even more significant, perhaps, are the advantages of well-judged partnerships, noting that ‘well-judged’ is the crucial word in this sentence.^5^

### Social progress and religious diversity

Diversity and democracy constituted the second level of the ascending scale. The two are related but the emphasis here will be on the former in line with the work of the University of Bristol’s Centre for the Study of Ethnicity and Citizenship. The section in question started by indicating the state of religious diversity in selected global regions; it continued by suggesting different ways in which these complex trends can be managed. Interestingly this turns the argument full circle in that some, if not all, of our suggestions for the good management of diverse religions were drawn from the work of Tariq Modood and colleagues. What better place than this special issue to acknowledge our debt?

In terms of both mapping and management, the summary account that follows draws very directly from the examples set out not only in the published version of ‘Religions and Social Progress: Critical Assessments and Creative Partnerships’ (2018), but in a longer version of the chapter housed on the IPSP website, which contains a series of extended case studies.^6^ Both versions contain helpful graphics.

### Mapping diversity

The starting point is clear: the presence of religious diversity across the globe is far from uniform (Grim, 2015). In most parts of the world diversity is growing, but in others it is declining, and in yet others it remains relatively stable. The following examples illustrate all three patterns, articulating the wide variety of reasons for both growth and decline over the period under review (1970–2015).

In the first category can be found societies that have been relatively stable for a longish period of time, but which are now becoming more religiously diverse. Western Europe is an obvious and high-profile example. Here there has been a significant and economically motivated upturn in immigration since the mid-twentieth century, bringing not only substantial numbers of Christians from the global South but a growing Muslim presence to societies that are themselves increasingly secular. The political consequences of this combination are considerable, but are experienced differently in different nation-states, reactions largely determined by the long-term settlements between church and state found in each case.^7^ Coterminous with this transformation, though much less noticed until the late twentieth century, has been the diversification *within* the major world faiths. The fragmentation of Latin American Christianity offers an excellent example, in which an almost uniformly Catholic global region is now experiencing the rapid growth of Protestantism, mostly in its Pentecostal forms. As in Europe, there is variation from country to country, but the changing nature of the continent overall – including the effects on Catholicism itself – reflects a wider global movement.^8^

Rather different are those parts of the world which were sites of aggressive and politically motivated secularization for most of the twentieth century but which are now experiencing religious restoration and growth. Since 1989, formerly hegemonic Orthodox churches have reasserted themselves strongly in Russia and Eastern Europe, but at the expense very often of minority religions (see the relevant chapters in Davie and Leustean, 2021). In China, the process is more complex. Not only does the Communist Party in China remain resolutely atheist, but its attitudes toward religions deemed ‘foreign’ are different from its dealings with Confucianism, Daoism, or Buddhism. Non-Chinese religions – notably Islam and Christianity – are increasingly seen as threats to national unity. The tragedy befalling the Muslim Uyghurs is widely publicized, far more so than it was when the IPSP chapter was drafted.^9^

A third set of cases is located in parts of the world where religious diversity most certainly exists but is nothing new. In Southeast Asia, for example, there has been little overall change in the period under review. That said, what might be called ‘constitutive’ diversity continues to evolve as migration – at times propelled by repression – moves religious traditions along with people. It is equally clear that colonialism altered the religious ecology in this part of the world (and elsewhere) in ways that can still be seen. The United States could hardly be more different, but it too is a society built on diversity, as wave after wave of migrants found their way there – initially across the Atlantic and more recently from very different parts of the world. Diversity is part of American self-understanding: individually, collectively and constitutionally. In recent years, however, the challenges have included the accommodation of faiths other than Christian, especially Islam – a politically sensitive step for some Americans.

The final group of cases reminds us that increasing, or even persistent, diversity is not uniformly the case; the reverse process also occurs, but not always for the same reason. In large parts of Africa, for instance, there is decreasing diversity due to a ‘modernizing’ process that encourages adherence to ‘world’ religions rather than to a multitude of local traditional faiths. Once again, however, careful attention should be paid to the details of each country – they are far from uniform. The Middle East, conversely, is characterized by decreasing diversity largely because of conflict and dispersion. The displacement of historic religious communities, long at home in the region, is a recent, continuing and tragic phenomenon.

The significance of mobility and migration – for whatever reason – pervades this field and forms a bridge to the material on the management of diversity. Unsurprisingly migration was a theme running right through the work of the IPSP given that the presence of migrants and the tensions surrounding them have become critical flashpoints in the twenty-first century, challenging societies to develop effective modes of reception and governance. Religion is a significant variable at every point, but it was not always recognized by our colleagues.

There exists, however, a distinguished body of research on the multifaceted relationship between religion and migration (see, for example, Warner and Wittner, 1998; Beckford, 2016), in which one theme stands out: that is the presence of a two-way flow, often mediated by the communication technologies that link communities across territory. Religions inspire, manage and benefit from the migration process, but at the same time beliefs, identities and practices are shaped and reshaped by the associated dislocating of populations. Take, for example, the evolution of religions that are ‘traditionally’ linked to particular global regions or national contexts. What happens when members of a religious majority learn to live as a minority in a new place, in which culture and religion are no longer interrelated? Equally significant are the currents that feed back into the country of origin and their effects on the home community. Two very different examples of diasporas and their influence can be found in Stoeckl’s work on the Orthodox Church (Stoeckl 2014) and Sinha’s account of the Hindu diaspora (Sinha 2019).

The role of religion in the reception of migrants is part and parcel of the same process and is the subject of Part V of Beckford’s first volume (2016). A particularly instructive illustration can be found in Margarita Mooney’s (2013) study of Haitian immigrants in three very different places: Miami, Montreal and Paris. Mooney notes that the differentiation between religion and the state in the United States (a structural variable) allowed faith-based organizations to assist and advocate for Haitians in Miami. In contrast, both Quebec (characterized by secular nationalism) and France (dominated by a more assertive secularism) discouraged community organizations based on religious or ethnic identifications. Mooney found that the greater scope for action allowed to the primarily religious mediating organizations established by the Haitian community in Miami more effectively assisted the reception of newcomers than was possible in either Quebec or France. Thus, macro, meso and micro levels are brought together in the understanding of religion as a crucial variable in the successful resettlement of migrants.

Religion, however, never stands alone. It is part of a bigger picture which must be approached contextually. Indeed, the possibility of changing or leaving one’s religion – or the country in which it is embedded – can by no means be taken for granted. For David Martin (2013), the big contrast lies between transnational voluntarism at one end of the spectrum, and forms of religion based on a closed market and which regard certain territories as their sacred preserve at the other:

The global variations run along a scale from North America, where it [the exercise of choice] is normal, to Western Europe and Australasia, where it is accepted but not all that frequent, to the Arabian Peninsula, which is by definition Islamic territory where even foreigners cannot establish their own sacred buildings. (Martin, 2013: 185)

Circumstances most definitely alter cases and will continue to do so.

### Managing diversity: multiculturalism and secularism

As set out in Chapter 2 of the IPSP report, social progress depends on establishing civil societies where people of diverse heritage can not only work and live together, but flourish in each other’s company. To achieve such a goal, each society must find a way forward within the parameters set by its past. In the religion chapter of the IPSP report, we set out two ways of thinking about this: sets of ideas captured by the terms ‘multiculturalism’ and ‘secularism’.

The idea of ‘multiculturalism’ had already been introduced in the chapter that preceded ours in Volume 3 of *Rethinking Society for the 21*^*st*^*Century* entitled ‘Social progress and cultural change’ (Bowen and Kymlicka et al., 2018). It is, of course, a term with multiple meanings. At one level, it is part of an expanding cultural market, allowing the discerning consumer to pick and choose from an increasing range of cultural goods (food, clothing, art, music, and so on). Very different in valance is the all-too-prevalent understanding of multiculturalism as an inherently divisive process that damages – necessarily – the dominant or host culture. Is it possible to find a more positive line of thinking? The authors of Chapter 15 offered the following solution: to grasp the building of a multicultural society as a process in which new ideas and new ways of doing things are constantly drawn into the mainstream, which is itself re-formed. ‘Instead of separate cultures – the foreign ones accommodated by the natural one – the culture of a place should be seen as tributaries that wash into a cultural river that is not given but continually constituted.’ (Bowen and Kymlicka et al., 2018: 631).

The religion chapter came to a similar conclusion drawing very directly on the work of Tariq Modood, a leading scholar in this field, taking as a starting point a summary article published in *The Guardian* (Modood, 2011). In this, Modood notes that the growing assertion of strongly held religious identities struck many as ‘too multicultural’ in the divisive sense, seeing such identities as very different from the friendly differences in music or food previously celebrated. The timing is important. The article was published against the background of a high-profile statement by the then UK Prime Minister indicating that multiculturalism had failed. Similar misgivings were felt in France and Germany – a situation that has become more rather than less pronounced in the subsequent decade. Undeniably it is sentiments such as these that have encouraged the far-right movements that are currently gaining purchase in many parts of Europe, exacerbated by the influx of migrants arriving from the Middle East, a flow that peaked in 2015. All too obvious in their rhetoric is the misappropriation of Europe’s Christian heritage in order to exclude rather than include, and to reject rather than welcome the stranger. Hennig and Weiberg-Salzberg (2021) bring together a series of thought-provoking case studies, some of them disturbing, that illustrate this point.

That said, many very varied actors have a part to play in finding a more constructive way forward. Among them are religious organizations at every level of society and the myriad ordinary individuals that inhabit them. Here, if used well, can be found a more positive resource. Drawing on the variables outlined above (pp. 3-4), this might include: the power of religious ideas to motivate, of religious practices to shape ways of life, of religious communities to initiate change, and of (local) religious leaders to legitimate calls to action. Here, in short, is the potential to promote what the chapter team termed ‘street-level ecumenism’, meaning by this the capacity not only to live and work in the same street or neighbourhood, but to thrive in in a diverse and plural environment.

Modood’s work has continued to evolve. A current (2018-22) European Union-funded project, entitled ‘Radicalisation, Secularism and the Governance of Religion: European and Asian Perspectives (GREASE), is helpful in terms of its global resonance.^10^ Specifically, the project asks what Europe can learn from other parts of the world about governing religious diversity? A ten-partner consortium has been established to probe this question in a wide variety of cultures, looking at the norms, laws and practices of diverse societies as they seek to integrate minorities and migrants. Attention will be paid to historical factors, not least the legacies of colonialism. What can Europeans (both scholars and policy-makers) learn from these data, as concerns about extremism grow in a continent that is currently experiencing the growth of religious radicalization alongside growing secularization – the paradox that defines twenty-first century Europe (see also Davie and Leustean, 2021). Also under review are the effects of increased connectivity, mobility and inter-dependence as well as widening inequalities and new forms of nationalism, all of which are relevant to the management of religious diversity.

Like multiculturalism, secularism has different meanings and it too is contentious. To resolve some of these differences, a complex terminology has evolved to distinguish forms of secularism that seek to accommodate difference from their more radical counterparts which exclude the presence of religion from the public square on principle. Rowan Williams (2012) terms the former ‘procedural’ secularism and the latter ‘programmatic’ secularism. Modood (2013) prefers ‘moderate’ and ‘radical’ as the operative terms, but the distinction is similar. Moderate secularism, moreover, is entirely compatible with multiculturalism.

That said, this relationship demands constant scrutiny and is interrogated in some depth in Modood’s (2019)*Essays on Secularism and Multiculturalism*, the subject of a number of symposia. In the first of a series of articles gathered in ‘A Conversation on Tariq Modood’s *Essays on Secularism and Multiculturalism’,* published in *Patterns of**Prejudice* (2021),^11^ Modood sets out his position:

… the first step of my argument is to show that Islamophobia is a form of cultural racism, and the next step is to show that anti-racism, whether in terms of a difference-blind neutral liberal state or in terms of active de-Othering, is not enough. We need a conception of equal citizenship that brings together the equality of same treatment with the equality of respect for difference, in short, a multiculturalism (Modood, 2021:2)

This respect for difference must moreover include religion – both Muslim and other – and that means revisiting the concept of political secularism. Not everyone will agree with Modood’s argument, but it captures very directly the spirit of the IPSP chapter. Religion, too often deemed part of the problem, must be seen as part of the solution – a pro-active partner in debates about diversity as a whole. How this will be achieved will vary from place to place. Modood is well-known for arguing that in the British – more especially English – case, the privileges of the established Church should not be abolished with a view to levelling the playing field. Such privileges should instead be shared. Minorities should be drawn into the privileges enjoyed by the mainstream, prepared at the same time to assume the associated responsibilities. Exactly how this will work will vary from case to case, depending on the size, experience and historical contribution of the minority in question.

In her path-breaking work on political liberalism, Cécile Laborde (2017) – a member of the IPSP Steering Committee – probes further. In *Liberalism’sReligion*, Laborde starts from the following question: should the liberal state necessarily be secular? In her response, she argues that there is indeed a minimal secularism – or separation between state and religion – that is required by the liberal state, but that secularism is more complex than is often thought. Specifically, it is incorrect to assume that liberal democracy requires the strict separation of state and religion that is found in the French or the US model. In reaching this conclusion, Laborde once again follows the argument of the IPSP chapter in that she underlines that religion is more – much more – than a statement of belief about what is true, or a code of moral and ethical conduct. Religion refers equally to ways of living, to political theories of justice, to modes of voluntary association and to vulnerable collective identities. Thus, Laborde disaggregates religion into its various dimensions, and in so doing dispenses with the Western, Christian-inflected conception of religion that liberal political theory relies on, particularly with reference to the separation between religion and state. As a result, there is considerably more variation in permissible state-religion arrangements than either secular liberals or religiously-minded liberals have often assumed – a flexibility that can be extended to non-Western societies. The Indian case is frequently cited in this respect: it offers an important and much-discussed example of the application of secularism beyond the West (Sinha, 2018).

### Religion and the global pandemic

Running through both the IPSP chapter and this article is an evident tension: is religion (and in particular religious diversity) perceived as a risk (in other words as part of the problem) or as a resource (as part of the solution)? Opinions differ and nowhere more so that in the efforts of policy-makers and those who advise them when faced with the ravages of COVID-19 as it spread across the world early in 2020.

In the UK, for example, it was quickly evident that certain communities were disproportionately vulnerable to the virus and were therefore at risk; equally problematic has been low vaccine-uptake in the same areas. The evidence for both statements is captured in a summary piece published in April 2021: ‘Over a year into the COVID-19 pandemic, ethnic minority communities in the UK and elsewhere continue to be affected by a disproportionate burden of COVID-19 associated morbidity and mortality’. And a line or two later: ‘The low COVID-19 vaccine uptake among “BAME” communities remains a priority for the government’ (Ala et al., 2021:1). The reasons for these disparities are many and varied and include socio-economic, ethnic and cultural variables as well as religious issues per se.

More interesting in terms of the argument presented here, however, is the co-authors’ awareness that a solution to this situation will remain elusive until the underlying issues are addressed. Keeping this in mind and recognizing the complexity of the situation and the need for contextual – indeed granular – analyses, these authors commend approaches which harness ‘the wide range of experience of multiple faith groups, prominent community leaders, and NHS staff regarding community engagement’, in order to develop and disseminate ‘culturally appropriate COVID-19 materials and interventions’ (Ala et al., 2021:1). The more that this happens, the more such communities become the solution rather than the problem, so much so that the diagrammatic presentation of the article’s recommendations on p. 2 – using the headings ‘covering messaging content, style and source’; ‘effective and relevant communication mediums’; ‘use of appropriate technology’; and ‘vaccine hesitancy’ – exhibits almost every recommendation made in the final section of the IPSP chapter. At the same time, it is an outworking of Modood’s stipulation that faith and faith communities are – and must remain – central to the understanding of difference in Western societies.

Such issues were brought to the fore in the section of the IPSP chapter on ‘Everyday wellbeing: Economy, education, health, and development’ – the penultimate stage of the ascending scale. As in the case study above, faith communities are all too often seen as an impediment to progress. There is no doubt that this happens, but it is not necessarily the case. The role of faith communities in finding a solution to the 2014-5 Ebola outbreak in West Africa offers an excellent illustration of positive action (Marshall and Smith, 2015). Sadly, collaboration was slow to develop: knowledge was lacking and partnerships delayed, hampering the fast and organized responses that were necessary in this case. Specifically, ‘Ebola’s close association with cultural and religious practices makes active community engagement especially important. Change of funeral practices was imperative to reversing the epidemic and religious leaders (modern and traditional, Muslim and Christian) had to be involved’ (Marshall and Smith, 2015: 2). Faith communities, these authors conclude, are much more likely to be part of the solution if they are included as full partners from the outset, bringing with them not only a resolution to the problem (in this case a change in funeral practice), but powerful communications networks and unrivalled local knowledge.

Katherine Marshall’s work is located in the Berkley Center for Religion, Peace, and World Affairs at Georgetown University which is currently co-ordinating a project entitled ‘Religious Responses to COVID-19’ – a collaborative endeavour that explores the responses of religious actors across the world to the pandemic. The aim is to discover and collate information that will enable policy-makers and practitioners in development to work with religious actors as together they respond to COVID-19. The growing database curated by the Berkley Centre is impressive.^12^

Such data sets are grist to the mill of the chapter that we were charged with writing for the IPSP as they are to this article: they demonstrate that religious communities, including minorities, can and do contribute to social progress across a wide variety of fields. Discovering the circumstances in which positive rather than negative outcomes are likely in this respect is central to the social-scientific task and guided our thinking for the IPSP. Equally important is the continuing work of the Centre for the Study of Ethnicity and Citizenship. It is a pleasure to contribute to a special issue commending the Centre’s past, present and future initiatives.

## Notes

1 Further details about the origins and goals of the IPSP are available at: https://www.ipsp.org/. For a short summary of the chapter on religion and the challenges confronted by the chapter team, see https://blogs.lse.ac.uk/religionglobalsociety/2019/01/a-lived-situated-and-constantly-changing-reality-why-religion-is-relevant-to-the-pursuit-of-social-progress/. All websites in this article were accessed on 02 February 2022.

2 The volumes covered different themes indicated in their subtitles. Volume 1: Socio-economic transformations; Volume 2: Political Regulation, Governance, and Societal Transformations; Volume 3: Transformations in Values, Norms, Cultures. Chapter 16 in Volume 3, entitled ‘Religions and social progress: Critical assessments and creative partnerships’ is the focus of this article.

3 The names and academic affiliations of the team were as follows: Coordinating Lead Authors: Grace Davie (University of Exeter, UK); Nancy T. Ammerman (Boston University, USA) Lead Authors: Samia Huq (BRAC University, Dhaka, Bangladesh); Lucian N. Leustean (Aston University, UK); Tarek Masoud (Harvard University, Kennedy School of Government, USA); Suzanne Moon (University of Oklahoma, USA); Jacob K. Olupona (Harvard Divinity School, USA); Vineeta Sinha (National University of Singapore); David A. Smilde (Tulane University, USA); Linda Woodhead (Lancaster University, UK), Fenggang Yang (Purdue University, USA) Contributing Author: Gina Zurlo (Boston University, USA)

4 See in particular the Pew-Templeton Global Religious Futures project, available at:http://www.globalreligiousfutures.org/

5 Partnerships emerged in every section of our chapter, but those is the field of health and welfare were particularly significant. See, for example, the series of articles published in *The Lancet* in July 2015, available at: https://www.thelancet.com/series/faith-based-health-care. The implications for the COVID-19 pandemic are multiple.

6 The extended chapter is available at: https://www.ipsp.org/resources. The case studies cover Europe, China, Singapore, the US, India and Venezuela. The expertise of particular members of the chapter team was fully exploited in these vignettes.

7 An up-to-date summary of this situation can be found in Davie and Leustean (2021), notably Chapter 32 and the case studies brought together in Part V.

8 The growth of Pentecostalism first in Latin America and then across the world has generated a huge literature most of which lies beyond the limits of this article. The work of the Pew Research Center (2006 and 2014) on both global Pentecostalism and the changing situation in Latin America offer useful starting points.

9 Fenggang Yang, a member of the chapter team, advised on the constantly changing situation in China. More information about his continuing work and China itself is available at: https://cla.purdue.edu/directory/profiles/fenggang-yang.html and https://globaleast.org/?_ga=2.191843052.772414818.1627464338-1672353312.1627464338

10 A project summary, including details of the consortium and their respective locations is available at: https://www.bristol.ac.uk/ethnicity/projects/grease/

11 Available at: https://www.tandfonline.com/doi/full/10.1080/0031322X.2020.1866873?src&fbclid=IwAR0iK28qMMCwzMc0NgJCVZ0fbrhS8JFh9vhL2ColuYWVMA-tOWcoui7WRFw

12 See https://berkleycenter.georgetown.edu/subprojects/religious-responses-to-covid-19
